# Overview of the Prevalence of Noncommunicable Chronic Diseases in the Brazilian Population Reporting Physical Inactivity

**DOI:** 10.3390/ijerph23080946

**Published:** 2026-07-23

**Authors:** Luana Alves Silva, Ana Clara de Brito Cintra, Ivan Gustavo Masselli dos Reis

**Affiliations:** Research Group on Technology Applied to Exercise Physiology—GTAFE, Health Data Science Postgraduate Program, Sao Francisco University, Braganca Paulista 12916-900, Brazil

**Keywords:** physical inactivity, noncommunicable chronic diseases, multimorbidity, public health

## Abstract

**Highlights:**

**Public health relevance—How does this work relate to a public health issue?**

- The prevalence of self-reported diabetes, hypertension, and obesity was assessed in a representative segment of the Brazilian population who reported not engaging in any form of leisure-time physical activity, not commuting by walking or cycling to work or school, and not performing physically demanding tasks at home or at work.
- The prevalence of multimorbidity involving diabetes, hypertension, and obesity was also evaluated among individuals who reported being physically inactive.

**Public health significance—Why is this work of significance to public health?**

- Diabetes was more frequently observed in combination with hypertension and obesity than as an isolated condition; obesity was the most prevalent noncommunicable chronic disease among young Brazilian adults, whereas hypertension was more prevalent among middle-aged adults.
- Physical inactivity was significantly associated with and predicted multimorbidity involving up to three noncommunicable chronic diseases and it was also significantly associated with obesity among middle-aged women and men, but only among young adult men.

**Public health implications—What are the key implications or messages for practitioners, policy makers and/or researchers in public health?**

- Public health policies targeting the prevention of obesity or hypertension may also have an indirect impact on the prevalence of diabetes.
- Strategies for the prevention of hypertension and obesity may be more effective if they are designed to be age-specific.

**Abstract:**

Exposure to physical inactivity (PI) may be a risk factor for the development of one or more noncommunicable chronic diseases (NCDs). The present study examined the prevalence of diabetes, hypertension, and obesity, as well as their various combinations, among the Brazilian population exposed to PI. A total of 323,448 Brazilian men and women aged 18 to 59 years from the 26 state capitals and the Federal District were contacted by telephone and answered questions regarding their physical activity (PA) and health conditions, including medical diagnoses of NCDs. Participants who reported not engaging in any type of PA at home, at work, during commuting, or during leisure time were assigned to the PI group, whereas the remaining participants comprised the control group. Physical inactivity was associated (*p* < 0.05) with a 16% and 28% higher prevalence of obesity among middle-aged female and male participants, respectively. It was also associated (*p* < 0.05) with a higher prevalence of combined diabetes and hypertension, diabetes and obesity, and hypertension and obesity, ranging from 49–273%, 60–113%, and 20–71%, respectively. Additionally, physical inactivity was associated (*p* < 0.05) with a 73–106% higher prevalence of the coexistence of diabetes, hypertension, and obesity.

## 1. Introduction

In the context of lifestyle and the prevention of noncommunicable diseases (NCDs), physical inactivity (PI) has a role as a central modifiable risk factor. Evidence suggests a 20–30% reduction in risk across more than 25 NCDs with the adoption of regular physical activity (PA) [1]. The evidence linking PA to the prevention of multiple diseases includes improved insulin sensitivity, reduced blood pressure, decreased adiposity, improved lipid profiles, lower systemic inflammation, enhanced immune function, improved mitochondrial function, and better regulation of gene expression related to metabolism and aging [2]. Regarding some of the most prevalent NCDs worldwide, PI may be responsible for approximately 6% and 7% of the burden of cardiovascular disease and type 2 diabetes, respectively [3].

According to the World Health Organization, a physically active adult accumulates at least 150 min of moderate-intensity or 75 min of vigorous-intensity PA per week [4,5]. This amount of PA can be achieved across different domains, including recreational and leisure activities, transportation (such as cycling, walking, or wheeling), and occupational or household tasks performed at school, work, or home [6]. Individuals who do not meet this minimum recommended level of PA may be at increased risk of developing one or more NCDs, as well as experiencing reduced life expectancy [5,7,8].

It has been estimated that approximately 31.3% of the global adult population does not meet these guidelines [9]. Although distinguishing between individuals who meet and those who do not meet the recommendations is useful for identifying the proportion of the population exposed to lower versus higher mortality risk [10,11], the physically inactive group is heterogeneous. Within this group, some individuals may be insufficiently active or close to achieving the recommended minimum level of physical activity, whereas others may be completely inactive and therefore fully exposed to the risks associated with physical inactivity [12].

The prevalence of individuals who do not engage in moderate- to vigorous-intensity physical activity during leisure time, commuting, occupational activities, or household tasks, as well as their risk of developing noncommunicable chronic diseases (NCDs), remains poorly characterized. This study assessed the prevalence of diabetes, hypertension, and obesity and examined their associations with physical inactivity (PI) in a representative sample of the Brazilian population. In addition, the prevalence of coexisting diabetes and hypertension, diabetes and obesity, hypertension and obesity, and the simultaneous presence of diabetes, hypertension, and obesity was evaluated, together with their associations with PI. The predictive value of PI for healthy status and the presence of one or more NCDs was investigated using univariate and multivariate logistic regression models adjusted for age, sex, smoking status, alcohol consumption, and PI. Furthermore, longitudinal analyses were conducted to examine trends in the odds of each outcome over the past one and a half decades.

## 2. Materials and Methods

### 2.1. Type of Study and Data

This is a retrospective, cross-sectional observational study. The data were obtained from the public database Vigilância de Fatores de Risco e Proteção para Doenças Crônicas por Inquérito Telefônico (VIGITEL), which is maintained by the Brazilian federal government [13]. Telephone numbers were randomly sampled for each of the 26 state capitals and the federal capital, and participants who consented to take part in the government survey were interviewed by telephone. The sex, age, and education distributions of the samples were weighted to match the official population estimates for each city in the respective year [13].

### 2.2. Participants

Participants were recruited from 2009 to 2024. The sample consisted of Brazilian adults of both sexes, aged 18 to 59 years (older individuals were excluded). Participants answered several questions regarding their sociodemographic characteristics, lifestyle, levels of PA, nutrition, health status and medical diagnosis. Only those who provided valid responses to all questions included in the study were considered eligible. The English translations of all questions and response options analyzed in this study are presented in Table 1 and Table 2. No post-imputed or post-processed variables from the VIGITEL database were used in the present study. More information on VIGITEL procedures, the sampling process, and the complete questionnaire is publicly available online at: https://svs.aids.gov.br/daent/cgdnt/vigitel/ (accessed on 10 July 2026).

**Figure 1:**
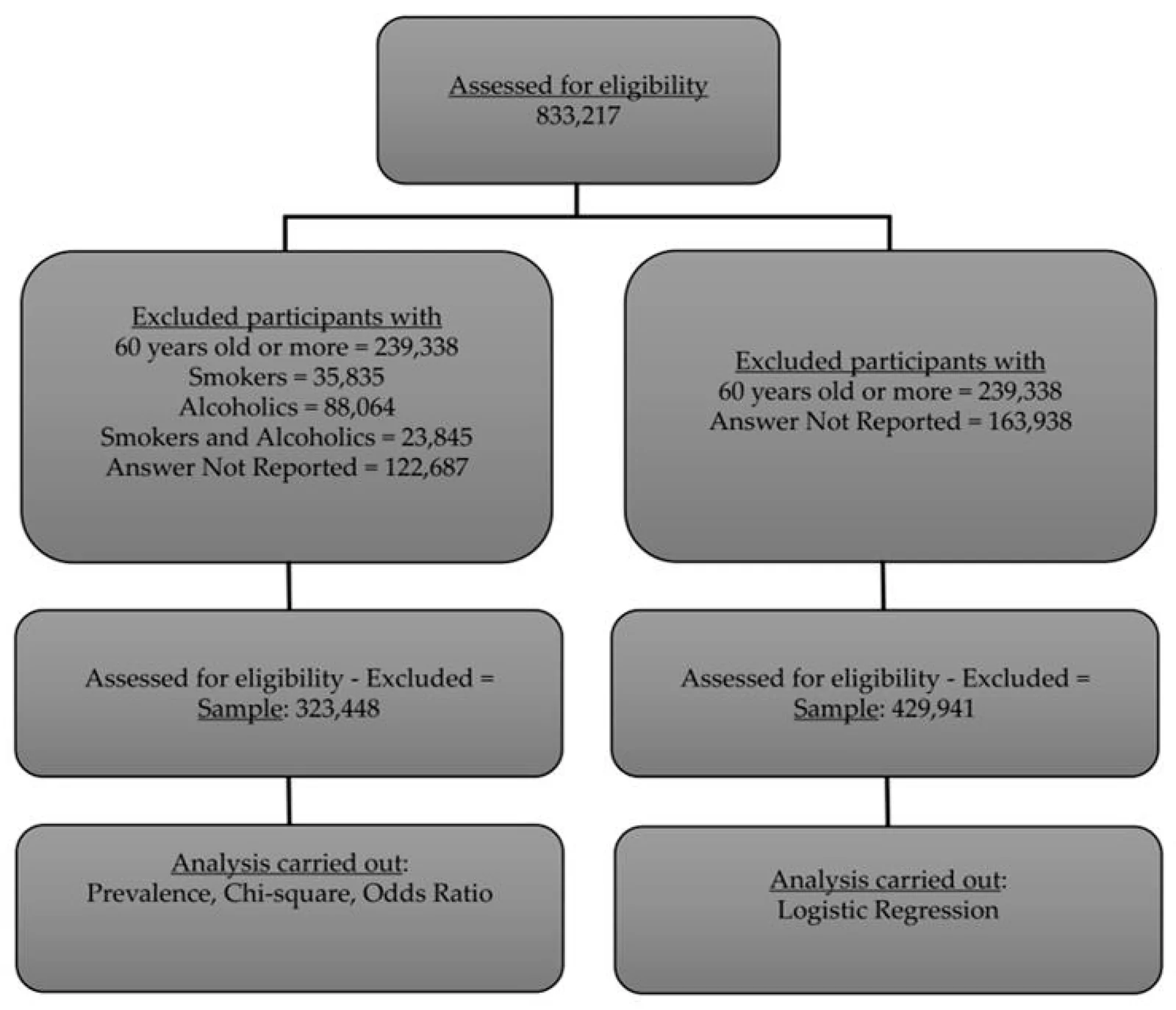
Initial and final sample sizes, showing the number of participants included after exclusions based on age, lifestyle, or missing information required for the study analysis.

The sample was segmented differently according to the statistical approach employed. For analyses comparing the prevalence of healthy status and NCDs between physically inactive and control participants, as well as for chi-square analyses and time-trend analyses using odds ratios, the sample was stratified by age and sex to better isolate the effect of PI. In addition, participants aged 60 years or older were excluded. Lifestyle-related exclusion criteria included current smoking and the reporting of at least one episode of excessive alcohol consumption per month, defined as the consumption of five drinks for men and four drinks for women on a single occasion. Participants with missing data for any variables required for group assignment, including physical activity level or the NCDs described in the following subsection, were also excluded. For the logistic regression analysis, a larger sample was retained to allow smoking status and alcohol consumption to be included as predictor variables in the univariate and multivariate models. Under this approach, only participants aged 60 years or older and those with incomplete responses were excluded (Figure 1).

### 2.3. Group Assignment

Participants were classified into either the physically inactive group or the control group based on their level of PA. To be assigned to the inactive group, participants were required to respond negatively to all six questions regarding engagement in physical activity across different contexts, including recreational and leisure activities, transportation (e.g., walking or cycling), and daily occupational tasks at school, work, or home. To be assigned to the control group, participants were required to report engaging in any amount of physical activity in their daily routine (Table 1).

Participants were classified into one of the following categories according to the self-reported presence of NCDs: healthy, diabetes, hypertension, obesity, diabetes and hypertension, diabetes and obesity, hypertension and obesity, or diabetes, hypertension, and obesity. To be assigned to the healthy group, participants were required to report no prior medical diagnosis of diabetes or hypertension and to have a body mass index (BMI) below 30 kg/m^2^, calculated from self-reported weight and height. Otherwise, participants were assigned to the single-NCD group if they reported a prior medical diagnosis of diabetes or hypertension or were classified as obese, or to the multiple-NCD group if they presented with two or all three of these conditions (Table 2).

### 2.4. Statistical Analyses

Statistical analyses were conducted using the Jupyter Notebook 3.6.3 (https://jupyter.org/) development environment and Python 3.11.3 (https://www.python.org/) as the programming language to calculate chi-squared test values and adjusted Pearson residuals for interpreting contingency tables. For the control and inactive groups, the prevalence of being healthy, having an isolated NCD, or having a combination of two or all three NCDs was calculated as a percentage of the total number of participants within a group. The prevalence ratio was calculated by dividing the prevalence in the inactive group by the corresponding prevalence in the control group. Pearson residuals were calculated as the difference between the observed and expected frequencies divided by the square root of the expected frequency. To facilitate comparisons across cells, the Pearson residuals were further standardized by their estimated standard deviation, yielding adjusted Pearson residuals (also referred to as adjusted standardized residuals). These residuals account for the row and column marginal proportions and are approximately normally distributed under the null hypothesis of independence [14]. To control for multiple comparisons across the cells of each contingency table, the significance level was adjusted using the Bonferroni correction by dividing the nominal significance level (α = 0.05) by the total number of cell comparisons. Adjusted Pearson residuals were considered statistically significant when their absolute values exceeded the Bonferroni-adjusted critical value derived from the standard normal distribution, thereby controlling the family-wise Type I error rate [14]. Associations between isolated noncommunicable chronic diseases (NCDs), combinations of two or three NCDs, and physical activity (PA) levels were assessed using Pearson’s chi-square test for each sex and age subgroup. Effect size was quantified using Cramér’s V, calculated as the square root of the chi-square statistic divided by the subgroup sample size. Because all contingency tables were 8 × 2, the smaller table dimension was equal to 2, resulting in a denominator term of k – 1 = 1 [15]. For the time-trend analysis, the odds of each outcome were calculated as the ratio of the number of individuals with the outcome to the number of individuals without the outcome. Univariate and multivariate logistic regression analyses were performed using the logit function from the Statsmodels library [16]. The estimated log-odds coefficients were exponentiated using Euler’s number (e) to obtain the odds associated with each outcome.

## 3. Results

Descriptive statistics of age, weight, or height between the control and inactive groups can be found in Table 3.

**Figure 2:**
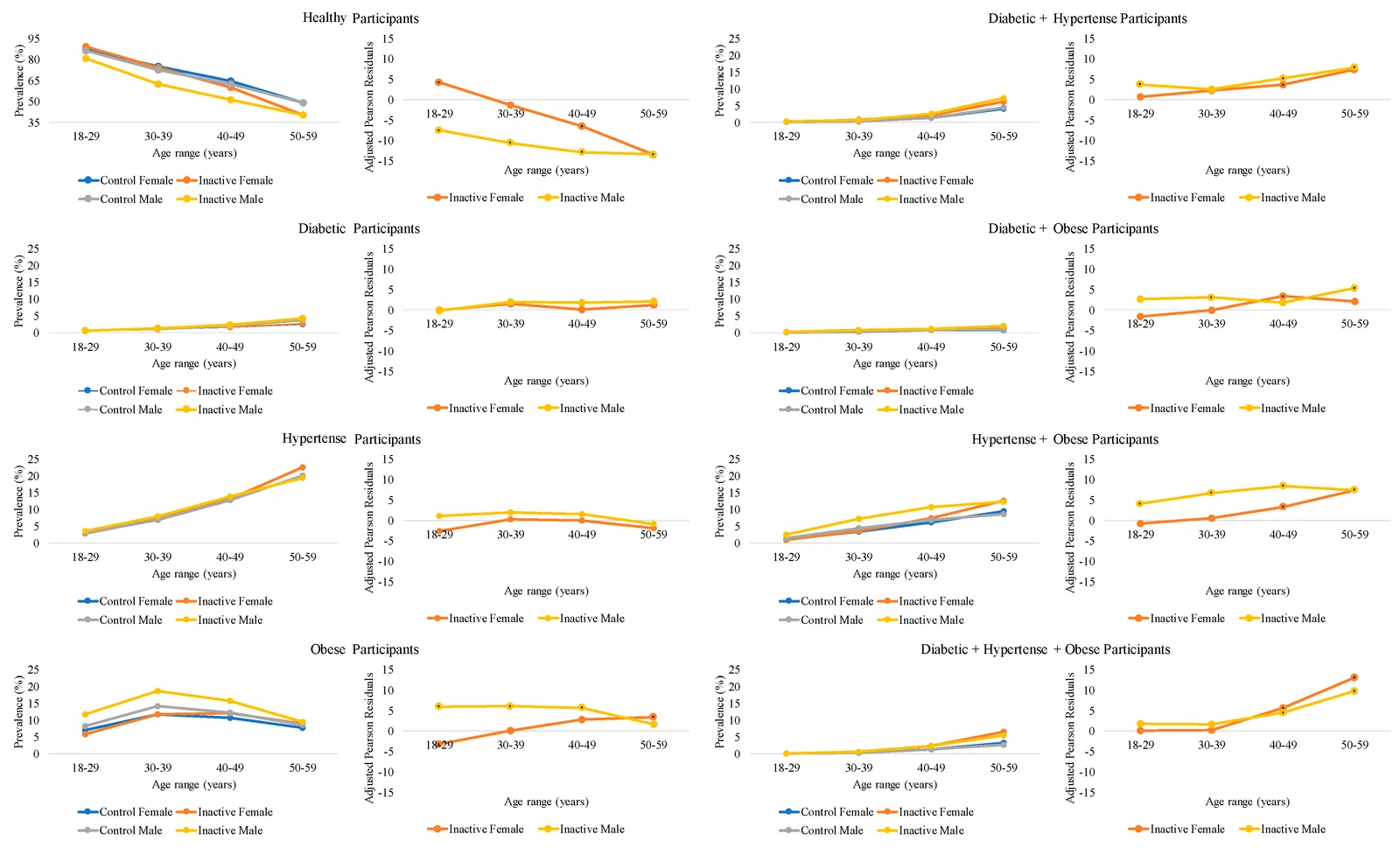
Prevalence of noncommunicable chronic diseases across age groups and physical activity levels in female and male participants. Adjusted Pearson residuals are shown for the inactive female and male groups. Black dots denote residuals greater than 2.94 or less than −2.94, indicating significant deviations from expected values at the Bonferroni-corrected significance level (*p* < 0.003125). No data were available from the VIGITEL dataset for 2022.

The level of physical activity was associated with changes in the prevalence of being healthy and obesity in the female participants aged 18 to 29 years (Table 4 and Figure 2). Among the female participants aged 30 to 39 years, no significant association between the level of physical activity and any NCDs was found (Table 4 and Figure 2). Among participants aged 40 to 49 years, the prevalence of healthy individuals was reduced in both inactive females and males (Table 4 and Table 5 and Figure 2). Similarly, physical inactivity was associated with an increased prevalence of all possible combinations of diabetes, hypertension, and obesity in both sexes, except for the combined presence of diabetes and obesity in male participants (Table 4 and Table 5 and Figure 2). In participants aged 50 to 59 years, hypertension prevalence was not associated with the level of physical activity in either females or males (Table 4 and Table 5 and Figure 2). Additionally, diabetes and the combined presence of diabetes and obesity were not associated with physical activity levels in female participants (Table 4 and Figure 2).

While the prevalence of being healthy decreased, the prevalence of obesity and the co-occurrence of obesity and hypertension increased among the inactive male participants aged 18 to 29 years (Table 5 and Figure 2). In contrast to female participants of the same age, significant associations were found among inactive male participants aged 30 to 39 years for the prevalence of being healthy, obesity, the co-occurrence of diabetes and obesity, and the co-occurrence of hypertension and obesity (Table 5 and Figure 2).

In male participants aged 50 to 59 years, health status and all combinations of diabetes, hypertension, and obesity were associated with levels of physical activity, including the presence of all three conditions (Table 5 and Figure 2).

Multivariate logistic regression models including age, sex, smoking status, alcohol consumption, and PI as predictors consistently showed higher pseudo-R^2^ values than the corresponding univariate models, regardless of the outcome considered. In these multivariate models, sex, smoking status, and PI exhibited similar predictive weights (exponentiated β values) for the isolated presence of diabetes, whereas age, smoking status, and alcohol consumption showed the greatest predictive weights for hypertension, and sex, alcohol consumption, and PI showed the greatest predictive weights for the isolated presence of obesity. Notably, PI was the strongest predictor of health status (lowest exponentiated β value) and of all combinations of co-occurring diabetes, hypertension, and obesity, including the simultaneous presence of all three comorbidities (Table 6).

**Figure 3:**
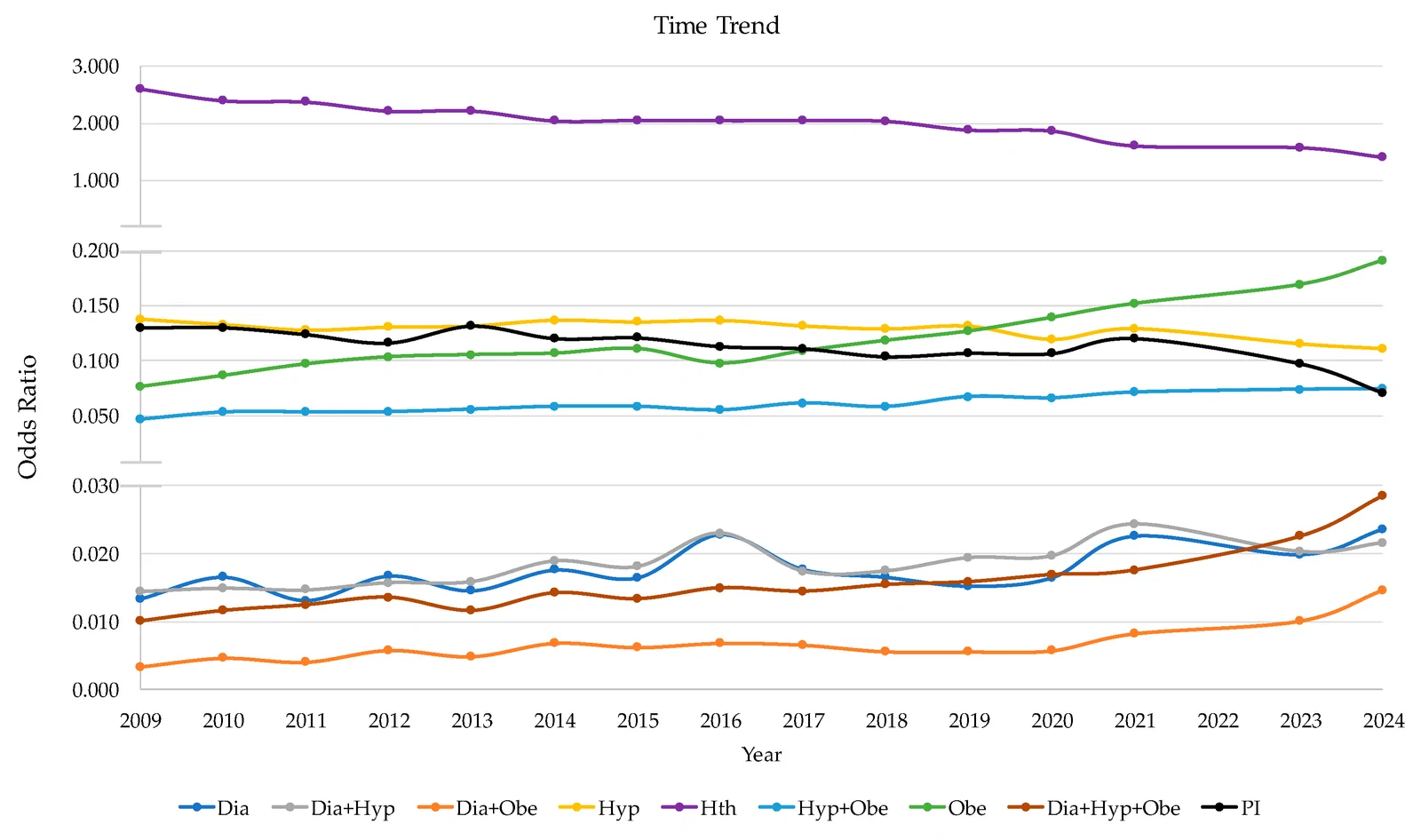
Odds of being healthy or having one or more noncommunicable chronic diseases over time. Abbreviations: Dia, diabetes; Hth, healthy; Hyp, hypertension; Obe, obesity; PI, physical inactivity. No data were available from the VIGITEL dataset for 2022.

The longitudinal analyses of the absolute odds of being healthy or having one or more NCDs indicated that the proportion of healthy individuals declined over the past one and a half decades. This trend was accompanied primarily by an increase in the absolute odds of obesity occurring in isolation, followed by diabetes. In contrast, hypertension and the presence of two or all three NCDs exhibited less absolute variation over time than the previously mentioned outcomes. However, when relative percentage changes were considered, all NCD-related outcomes appeared to increase over the study period, with the exception of hypertension and physical inactivity, which exhibited lower values in the final year of the study than in the first year analyzed. The prevalence of PI was also lower in the final year of the study than in the first year analyzed (Figure 3).

## 4. Discussion

The present study analyzed the association between PI and the prevalence of diabetes, hypertension, and obesity in a large sample of the Brazilian population. The prevalence of healthy participants was inversely associated with PI across all age ranges among male subjects and among female subjects aged 40 to 59 years (Table 4 and Table 5, Figure 2). Moreover, visual inspection of the time trends indicated a decline in the odds of being healthy over the past one and a half decades (Figure 3). Among the three NCDs analyzed, obesity was the most prevalent among young adults, followed by hypertension, whereas the inverse pattern was observed among middle-aged adults, with hypertension being more prevalent than obesity (Table 4 and Table 5, Figure 2). Logistic regression models revealed that PI was the most influential predictor of healthy status and of the co-occurrence of diabetes, hypertension, and obesity, including the simultaneous presence of all three comorbidities (Table 6).

While the bulk of epidemiological studies indicates that PI is associated with a higher risk of developing diabetes [17], the prevalence of diabetes in this study was not associated with PI across any age range in either female or male participants, including those aged 50 to 59 years (Table 4 and Table 5, Figure 2). However, the results of the univariate and multivariate logistic regression models showed a significant association between PI and diabetes (Table 6). One possible explanation is that controlling for confounders such as obesity and alcohol use may have weakened the observed association between diabetes and PI. Indeed, a significant association between PI and the co-occurrence of diabetes and obesity was observed across different age ranges in both female and male participants. Additionally, cross-sectional studies may yield null associations because they do not account for disease onset and are susceptible to reverse causation, whereby individuals may increase their PA after being diagnosed with the disease.

A previous study using the VIGITEL database has already demonstrated an association between PI and diabetes, hypertension, and obesity [18]. However, this may be the first study to examine the overlapping prevalence of diabetes, hypertension, and obesity in a large, nationally representative sample of the Brazilian population. The most prevalent cluster was hypertension and obesity, followed by diabetes and hypertension. Among individuals aged 40 to 59 years, diabetes was more frequently observed in combination with hypertension or with both conditions simultaneously than as an isolated condition, regardless of physical activity level or sex; the only exception was the co-occurrence of diabetes and obesity. In contrast, hypertension was more often observed as an isolated condition, regardless of physical activity level age and sex, whereas obesity was more frequently found as an isolated condition among individuals aged 18 to 49 years and in combination with hypertension after age 49 in both female and male participants. Indeed, a study conducted in a Spanish sample (Madrid) showed that 94.4% of participants with diabetes had multimorbidity [19]. In the Brazilian context, these findings suggest that public health policies targeting hypertension and obesity may also have an indirect impact on diabetes prevalence. Furthermore, strategies for hypertension prevention may be applicable across all ages and sexes, whereas approaches to obesity prevention may need to be age-specific.

Regarding the co-occurrence of comorbidities, the highest prevalence ratios and significant Pearson residuals were observed for the associations between PI and the prevalence of two or all three comorbidities (Table 4 and Table 5, Figure 2). Similar findings were obtained in the logistic regression models, in which PI emerged as the weighted predictor among all predictors included (Table 6). These results suggest an important role of PI in the epidemiology of multimorbidity. Evidence indicates that the accumulation of two or more NCDs is associated with an increased risk of mortality [20]. Therefore, PI may play an important role in the development of multimorbidity and may contribute indirectly to increased mortality risk, representing a key modifiable factor for public health interventions and health promotion policies.

Similar to the presence of diabetes as an isolated health condition, hypertension seems to be a comorbidity not impacted by PI, even with the visually prevalence increase in function of the age and although it was the most prevalent NCD in the middle-age adults (Table 4 and Table 5, Figure 2). The prevalence of hypertension in the Brazilian population was reported as 22.8% in a previous study based on the VIGITEL database [21]. This value is higher than the prevalence observed in both the Ctl and physically inactive groups in the present study. However, the earlier analysis included individuals older than 59 years. Arterial blood pressure is strongly associated with age [22,23]. Therefore, the decision to include only participants aged 59 years or younger (as individuals aged 60 years and older are considered elderly in Brazil) was intended to minimize the influence of advanced age on the dependent variables (health status) and better isolate the effect of the PI.

It has been reported that the prevalence of diabetes and hypertension among Brazilians with obesity is more than twice that observed among individuals who are of normal weight or underweight [24]. Part of this increased prevalence may be attributable to PI, as the present study found statistically significant associations ranging from 60% to 113% and from 20% to 71% in the prevalence of combined diabetes and obesity and hypertension and obesity, respectively (Table 4 and Table 5). Although the prevalence of isolated obesity was less affected by PI (statistically significant associations ranging from 13% to 41%) compared with multimorbidity conditions, it remains a public health concern, as obesity is associated with increased utilization of healthcare services in Brazil [25].

There is evidence that BMI calculated from self-reported height and weight may underestimate obesity prevalence [26]. This may occur because individuals tend to overestimate their height and underestimate their weight, leading to lower BMI values [27]. Lower estimates of obesity prevalence from survey data, compared with objectively measured data, have been consistently reported in large population-based studies [28]. Another limitation related to obesity is the classification of individuals with a BMI of 25.0–29.9 kg/m^2^ as healthy, although they may be at an increased risk of developing cardiometabolic diseases [29]. Considering that the VIGITEL data on obesity prevalence have not been validated using external tools or measurements, this bias may still have affected the prevalence estimates in the present study.

While it is possible that PI leads to a higher incidence of NCDs, reverse causation is also plausible, as individuals with NCDs may reduce their PA due to health-related limitations. Additionally, when an outcome is common, odds ratios can overestimate the magnitude of an association if they are interpreted as risk ratios or prevalence ratios This occurs because odds and probabilities diverge as the outcome prevalence increases [30]. Therefore, epidemiological studies based on observational approaches may be biased by reverse causation and statistical limitations, particularly in the context of chronic diseases [3]. Consequently, it cannot be definitively determined whether the associations between NCDs and PA levels observed in the present study represent a cause or a consequence.

Regarding the self-reported level of physical activity, the VIGITEL questionnaire relies on six questions addressing engagement in physical activity across different domains, such as domestic routines, occupational activities, leisure time, and urban commuting. It is likely that participants who report not engaging in physical activity in any of these domains are physically inactive. However, some questions may be interpreted inconsistently because they rely on subjective terms such as “a lot” or “a little,” which may not accurately reflect physical inactivity due to the absence of intensity-specific measures (Table 1). Additionally, the subjective nature of the responses cannot be fully accounted for, as VIGITEL data have not been validated against other instruments or objective measurement tools.

Although participants were asked about prior medical diagnoses, another limitation is the reliance on self-reported data regarding the presence of NCDs. While the sample was stratified by sex and age, and lifestyle factors such as smoking and abusive alcohol consumption were considered as exclusion criteria in the study design, several other potential confounding factors were not accounted for in the present study, including diet, education, income, geographic region, and genetic factors. Furthermore, the results may be influenced by temporal trends (Figure 3), given that the study is based on 15 years of annual cross-sectional data collection. These VIGITEL data collection limitations should be acknowledged when interpreting the present results and conclusions.

Because Cramer’s V summarizes the overall strength of association across the entire table, across the subgroups of sex and age, the values for the association between PI and the NCDs were weak or insignificant, even when adjusted Pearson residuals were high, indicating that certain prevalences occurred more or less often than expected. This may be due to a large sample size, a small number of cells with notable deviations, or deviations that are small relative to the entire table.

## 5. Conclusions

The main finding of this study is that PI exposure is associated with prevalence and is a significant predictor of obesity among middle-aged Brazilian men and women. The strongest associations were observed in overlapping comorbidity combinations, particularly diabetes and hypertension, hypertension and obesity, and the coexistence of all three conditions.

## Figures and Tables

**Table 1:** Questions on participants’ physical activity levels and the specific combination of answers used to assign them to the control or low levels of physical activity groups.

Question	Alternatives	Answer	Group Allocation
In the last three months, have you engaged in any type of physical activity?	1—Yes	1	Control
	2—No	2 ^#^	Inactive
To go to or return from your workplace, do you travel by walking or by bicycle?	1—Yes, the entire route	1 or 2	Control
	2—Yes, part of the route		
	3—No	3 ^#^	Inactive
To go to or return from a course or school, do you travel by walking or by bicycle?	1—Yes, the entire route	1 or 2	Control
	2—Yes, part of the route		
	3—No	3 ^#^	Inactive
At your job, do you walk a lot?	1—Yes	1	Control
	2—No	2 ^#^	Inactive
At your job, do you carry heavy loads or perform other physically demanding activities?	1—Yes	1	Control
	2—No	2 ^#^	Inactive
Who usually does the house cleaning in your home?	1—I do it alone	1 or 2	Control
	2—I do with another person		
	3—Another person	3 ^#^	Inactive

^#^ Mandatory for the respective group allocation.

**Table 2:** Questions on participants’ health status and the specific combination of answers used to assign them to the health or noncommunicable chronic diseases groups.

Question	Alternatives	Answer	Group Allocation
Has any doctor ever told you that you have diabetes?	1—Yes	1	Dia or Dia + Hyp or Dia + Obe or Dia + Hyp + Obe
	2—No	2 or 3 ^#^	Hth
	3—I do know		
Has any doctor ever told you that you have high blood pressure?	1—Yes	1	Hyp or Dia + Hyp or Hyp + Obe or Dia + Hyp + Obe
	2—No	2 or 3 ^#^	Hth
	3—I do not know		
Even if it is an approximate value, do you know your weight?	Only values ≥ 30 and <300 kg are accepted *	BMI ≥ 30	Obe or Dia + Obe or Hyp + Obe or Dia + Hyp + Obe
		BMI < 30 ^#^	Hth
Even if it is an approximate value, do you know your height?	Only values ≥ 120 and <220 cm are accepted *	BMI ≥ 30	Obe or Dia + Obe or Hyp + Obe or Dia + Hyp + Obe
		BMI < 30 ^#^	Hth

^#^ Mandatory for the respective group allocation, * Used for calculating the body mass index as weight (kg) divided by the height (m) squared, BMI: body mass index, Dia: Diabetes, Hth: health, Hyp: hypertension, Obe: obesity.

**Table 3:** Average and standard deviation values of age, weight and height of female and male participants in each subgroup of age for the control and inactive groups.

		Age (Years)		Weight (kg)		Height (cm)	
	Age Range (Years)	Ctl	Inactive	Ctl	Inactive	Ctl	Inactive
Female	18–29	23.7 ± 3	23.0 ± 3	62.3 ± 12	60.8 ± 13	162.3 ± 7	162.3 ± 7
	30–39	34.5 ± 2	34.5 ± 2	66.8 ± 13	67.1 ± 14	161.5 ± 7	162.0 ± 7
	40–49	44.3 ± 2	44.3 ± 2	67.4 ± 13	69.2 ± 15	160.5 ± 7	160.8 ± 7
	50–59	54.3 ± 2	54.6 ± 2	67.4 ± 12	70.2 ± 15	159.3 ± 7	159.3 ± 7
Male	18–29	23.3 ± 3	23.7 ± 3	76.3 ± 14	77.2 ± 16	175.1 ± 7	174.5 ± 8
	30–39	34.5 ± 2	34.6 ± 2	81.6 ± 15	84.0 ± 18	174.0 ± 7	173.5 ± 7
	40–49	44.3 ± 2	44.4 ± 2	81.2 ± 14	83.8 ± 17	172.6 ± 7	172.4 ± 7
	50–59	54.2 ± 2	54.4 ± 2	79.5 ± 14	81.4 ± 17	171.3 ± 7	170.7 ± 7

Abbreviation: Ctl, control.

**Table 4:** Number of female participants by age range and health status or the presence of one or more noncommunicable chronic diseases, including prevalence values, chi-square test results, Adjusted Pearson residuals, and Cramer’s V for physically inactive and control groups.

n = 207,902	Female	Number (%)		Prevalence	Chi-Square		Residuals
Status	Age (Years)	Ctl	Inactive	Ratio	Ctl	Inactive	Inactive
Hth	18–29	35,903 (87.1%)	4831 (89.1%)	1.02	0.27 (1.2%)	2.04 (9.1%)	4.26 *
	30–39	33,484 (75.1%)	3293 (74.2%)	0.99	0.04 (0.5%)	0.41 (4.6%)	−1.35
	40–49	32,273 (64.7%)	2824 (60.0%)	0.93	1.30 (1.5%)	13.74 (15.4%)	−6.49 *
	50–59	25,322 (49.3%)	2523 (40.2%)	0.82	10.28 (2.7%)	84.28 (22.0%)	−13.52 *
Dia	18–29	280 (0.7%)	37 (0.7%)	1.01	0.00 (0.0%)	0.00 (0.0%)	0.03
	30–39	531 (1.2%)	65 (1.5%)	1.23	0.22 (2.5%)	2.26 (25.4%)	1.58
	40–49	828 (1.7%)	79 (1.7%)	1.01	0.00 (0.0%)	0.01 (0.0%)	0.09
	50–59	1310 (2.6%)	177 (2.8%)	1.11	0.18 (0.0%)	1.45 (0.4%)	1.29
Hyp	18–29	1461 (3.5%)	154 (2.8%)	0.80	0.80 (3.5%)	6.03 (26.9%)	−2.66
	30–39	3114 (7.0%)	316 (7.1%)	1.02	0.01 (0.1%)	0.10 (1.1%)	0.33
	40–49	6604 (13.2%)	623 (13.2%)	1.00	0.00 (0.0%)	0.00 (0.0%)	−0.02
	50–59	11,589 (22.6%)	1348 (21.5%)	0.95	0.30 (0.1%)	2.47 (0.6%)	−1.89
Obe	18–29	2912 (7.2%)	319 (5.9%)	0.83	1.11 (5.0%)	8.47 (37.8%)	−3.21 *
	30–39	5262 (11.8%)	526 (11.9%)	1.00	0.00 (0.0%)	0.01 (0.1%)	0.09
	40–49	5330 (10.7%)	568 (12.1%)	1.13	0.65 (0.7%)	6.88 (7.7%)	2.91
	50–59	3977 (7.7%)	563 (9.0%)	1.16	1.19 (0.3%)	9.72 (2.5%)	3.44 *
Dia + Hyp	18–29	67 (0.2%)	11 (0.2%)	1.25	0.05 (0.2%)	0.41 (1.8%)	0.69
	30–39	231 (0.5%)	35 (0.8%)	1.52	0.49 (5.5%)	4.95 (55.6%)	2.34
	40–49	675 (1.4%)	95 (2.0%)	1.49	1.16 (1.3%)	12.29 (13.8%)	3.69 *
	50–59	2152 (4.2%)	390 (6.2%)	1.49	5.69 (1.5%)	46.64 (12.2%)	7.40 *
Dia + Obe	18–29	79 (0.2%)	5 (0.1%)	0.48	0.31 (1.4%)	2.32 (10.3%)	−1.62
	30–39	194 (0.4%)	19 (0.4%)	0.98	0.00 (0.0%)	0.00 (0.0%)	−0.07
	40–49	377 (0.8%)	57 (1.2%)	1.60	0.96 (1.1%)	10.22 (11.5%)	3.36 *
	50–59	428 (0.8%)	69 (1.1%)	1.32	0.50 (0.1%)	4.14 (1.1%)	2.16
Hyp + Obe	18–29	484 (1.2%)	57 (1.1%)	0.90	0.07 (0.3%)	0.55 (2.4%)	−0.79
	30–39	1543 (3.5%)	161 (3.6%)	1.05	0.03 (0.3%)	0.29 (3.3%)	0.58
	40–49	3100 (6.2%)	350 (7.4%)	1.20	0.87 (1.0%)	9.21 (10.3%)	3.28 *
	50–59	4931 (9.6%)	789 (12.6%)	1.31	5.47 (1.4%)	44.80 (11.7%)	7.47 *
Dia + Hyp + Obe	18–29	59 (0.1%)	8 (0.2%)	1.03	0.00 (0.0%)	0.01 (0.0%)	0.08
	30–39	207 (0.5%)	22 (0.5%)	1.07	0.01 (0.1%)	0.08 (0.9%)	0.29
	40–49	665 (1.3%)	111 (2.4%)	1.77	2.7368 (3.1%)	28.9858 (32.6%)	5.67 *
	50–59	1692 (3.3%)	413 (6.6%)	2.00	18.06 (4.7%)	148.02 (38.6%)	13.13 *
Total	18–29	41,245 (100%)	5422 (100%)	-	2.61 (11.6%)	19.83 (88.4%)	
	30–39	44,566 (100%)	4437 (100%)	-	0.81 (9.1%)	8.09 (91%)	
	40–49	49,852 (100%)	4628 (100%)	-	7.6781 (8.6%)	81.3191 (91.4%)	
	50–59	51,401 (100%)	6272 (100%)	-	26.59 (12.3%)	189.09 (87.7%)	Craemer’s V
	18–29	46,667			22.44 (100%)		0.02
Ctl + Inactive	30–39	49,003			8.89 (100%)		0.01
Total	40–49	54,480			88.99 (100%)		0.04
	50–59	57,673			215.68 (100%)		0.09

* Adjusted Pearson residuals greater than 2.94 or less than −2.94 are significantly different from expected at a Bonferroni-corrected critical *p*-value of 0.003125. Abbreviations: Ctl, control; Dia, diabetic; Hth, healthy; Hyp, hypertense; Obe, obese.

**Table 5:** Number of male participants by age range and health status or the presence of one or more noncommunicable chronic diseases, including prevalence values, chi-square test results, Adjusted Pearson residuals, and Cramer’s V for physically inactive and control groups.

n = 115,546	Male	Number (%)		Prevalence	Chi-Square		Residuals
Status	Age (Years)	Ctl	Inactive	Ratio	Ctl	Inactive	Inactive
Hth	18–29	26,794 (86.2%)	2090 (80.9%)	0.94	0.61 (0.74%)	7.35 (8.9%)	−7.49 *
	30–39	16,175 (72.4%)	1682 (62.6%)	0.87	3.42 (2.6%)	28.47 (21.3%)	−10.55 *
	40–49	15,555 (62.5%)	1791 (51.2%)	0.82	7.91 (3.7%)	56.28 (26.1%)	−12.84 *
	50–59	12,351 (50.7%)	1610 (39.4%)	0.78	12.99 (3.9%)	77.50 (23.5%)	−13.33 *
Dia	18–29	189 (0.6%)	15 (0.6%)	0.95	0.00 (0.00%)	0.03 (0.0%)	−0.17
	30–39	209 (0.9%)	36 (1.3%)	1.43	0.43 (0.3%)	3.59 (2.7%)	2.02
	40–49	477 (1.9%)	83 (2.4%)	1.24	0.40 (0.2%)	2.83 (1.3%)	1.82
	50–59	893 (3.7%)	178 (4.4%)	1.19	0.64 (0.2%)	3.83 (1.2%)	2.15
Hyp	18–29	985 (3.2%)	92 (3.6%)	1.12	0.09 (0.1%)	1.05 (1.3%)	1.08
	30–39	1532 (6.9%)	212 (7.9%)	1.15	0.40 (0.3%)	3.32 (2.5%)	2.00
	40–49	3203 (12.9%)	483 (13.8%)	1.07	0.26 (0.1%)	1.81 (0.8%)	1.54
	50–59	4897 (20.1%)	795 (19.5%)	0.97	0.10 (0.0%)	0.60 (0.2%)	−0.93
Obe	18–29	2587 (8.3%)	304 (11.8%)	1.41	2.52 (3.0%)	30.32 (36.7%)	5.99 *
	30–39	3196 (14.3%)	503 (18.7%)	1.31	3.42 (2.6%)	28.43 (21.3%)	6.11 *
	40–49	3069 (12.3%)	553 (15.8%)	1.28	3.58 (1.7%)	25.45 (11.8%)	5.77 *
	50–59	2107 (8.7%)	387 (9.5%)	1.10	0.39 (0.1%)	2.35 (0.7%)	1.73
Dia + Hyp	18–29	29 (0.1%)	9 (0.4%)	3.73	1.05 (1.3%)	12.68 (15.3%)	3.71 *
	30–39	85 (0.4%)	19 (0.7%)	1.86	0.66 (0.5%)	5.51 (4.1%)	2.49
	40–49	344 (1.4%)	89 (2.5%)	1.84	3.34 (1.6%)	23.79 (11.0%)	5.25 *
	50–59	1090 (4.5%)	300 (7.4%)	1.64	8.48 (2.6%)	50.58 (15.4%)	7.88 *
Dia + Obe	18–29	23 (0.1%)	6 (0.2%)	3.14	0.53 (0.6%)	6.39 (7.7%)	2.63
	30–39	78 (0.4%)	20 (0.7%)	2.13	1.03 (0.8%)	8.56 (6.4%)	3.10 *
	40–49	210 (0.8%)	40 (1.1%)	1.35	0.39 (0.2%)	2.74 (1.3%)	1.78
	50–59	252 (1.0%)	82 (2.0%)	1.94	4.05 (1.2%)	24.19 (7.3%)	5.35 *
Hyp + Obe	18–29	458 (1.5%)	65 (2.5%)	1.71	1.28 (1.6%)	15.37 (18.6%)	4.11 *
	30–39	987 (4.4%)	197 (7.3%)	1.66	4.63 (3.5%)	38.57 (28.9%)	6.73 *
	40–49	1702 (6.8%)	379 (10.8%)	1.58	8.23 (3.8%)	58.53 (27.1%)	8.49 *
	50–59	2114 (8.7%)	503 (12.3%)	1.42	7.23 (2.2%)	43.16 (13.1%)	7.45 *
Dia + Hyp + Obe	18–29	18 (0.1%)	4 (0.2%)	2.67	0.26 (0.3%)	3.16 (3.8%)	1.85
	30–39	83 (0.4%)	16 (0.6%)	1.60	0.33 (0.2%)	2.73 (2.04%)	1.75
	40–49	338 (1.4%)	82 (2.3%)	1.73	2.48 (1.2%)	17.66 (8.2%)	4.52 *
	50–59	662 (2.7%)	229 (5.6%)	2.06	13.39 (4.1%)	79.91 (24.3%)	9.81 *
Total	18–29	31,083 (100%)	2585 (100%)	-	6.35 (7.7%)	76.4 (92.3%)	
	30–39	22,345 (100%)	2685 (100%)	-	14.32 (10.7%)	119.18 (89.3%)	
	40–49	24,898 (100%)	3500 (100%)	-	26.59 (12.3%)	189.09 (87.7%)	
	50–59	24,366 (100%)	4084 (100%)	-	47.3 (14.4%)	282.11 (85.6%)	Craemer’s V
	18–29	33,668			82.70 (100%)		0.05
Ctl + Inactive	30–39	25,030			133.51 (100%)		0.07
Total	40–49	28,398			215.68 (100%)		0.09
	50–59	28,450			329.39 (100%)		0.11

* Adjusted Pearson residuals greater than 2.94 or less than −2.94 are significantly different from expected at a Bonferroni-corrected critical *p*-value of 0.003125. Abbreviations: Ctl, control; Dia, diabetic; Hth, healthy; Hyp, hypertense; Obe, obese.

**Table 6:** Unadjusted and adjusted logistic regression models of healthy status and noncommunicable chronic diseases, adjusted for age, sex, smoking, alcoholism, and physical inactivity.

n = 429,941		Model 1 (Unadjusted)			Model 2 (Adjusted)		
Outcomes	Predictors	Exponentiated β (95%CI)	Standard Error	Pseudo-R^2^	Exponentiated β (95%CI)	Standard Error	Pseudo-R^2^
	Age (years)	0.9422 * (0.9416–0.9428)	0.0003	0.07567	0.9429 * (0.9423–0.9435)	0.0003	
	Sex (male)	0.8961 * (0.8836–0.9087)	0.0063	0.000066	0.9613 * (0.9490–0.9738)	0.0064	
Hth	Smoking	1.1347 * (1.1083–1.1617)	0.0080	0.00068	0.9337 * (0.9134–0.9546)	0.0078	0.07825
	Alcoholism	0.8645 * (0.8494–0.8798)	0.0105	0.00057	0.9764 * (0.9608–0.9921)	0.0138	
	Physical Inactivity	0.7457 * (0.7300–0.7618)	0.0074	0.001894	0.7160 * (0.7017–0.7306)	0.0082	
	Age (years)	1.0526 * (1.0503–1.0550)	0.0012	0.03131	1.0517 * (1.0494–1.0541)	0.0012	
	Sex (male)	1.0703 ^#^ (1.0203–1.1227)	0.0261	0.0001086	1.1913 * (1.1342–1.2513)	0.0306	
Dia	Smoking	1.2709 * (1.1785–1.3705)	0.0490	0.0005127	1.1746 * (1.0877–1.2685)	0.0479	0.03324
	Alcoholism	0.7102 * (0.6644–0.7592)	0.0242	0.001536	0.7377 * (0.6884–0.7905)	0.0269	
	Physical Inactivity	1.2703 * (1.1830–1.3641)	0.0462	0.0005750	1.1702 * (1.0893–1.2570)	0.0443	
	Age (years)	1.0670 * (1.0660–1.0680)	0.0005	0.07106	1.0670 * (1.0660–1.0680)	0.0005	
	Sex (male)	0.8837 * (0.8670–0.9008)	0.0086	0.0005242	0.9488 * (0.9298–0.9682)	0.0099	
Hyp	Smoking	1.2108 * (1.1741–1.2487)	0.0190	0.0004628	1.0528 * (1.0162–1.0836)	0.0140	0.07122
	Alcoholism	0.9090 * (0.8873–0.9312)	0.0112	0.0001983	1.0494 * (1.0260–1.0803)	0.0175	
	Physical Inactivity	1.0560 * (1.0246–1.0884)	0.0163	0.0000402	0.9755 (0.9457–1.0063)	0.0157	
	Age (years)	0.9999 (0.9991–1.0007)	0.0004	0.0000003	1.0009 * (1.0000–1.0017)	0.0004	
	Sex (male)	1.2282 * (1.2043–1.2527)	0.0123	0.001454	1.1870 ^•^ (1.1631–1.2113)	0.0124	
Obe	Smoking	0.9568 ^•^ (0.9241–0.9906)	0.0170	0.0000219	0.8806 * (0.8499–0.9124)	0.0162	0.002889
	Alcoholism	1.2729 * (1.2434–1.3030)	0.0152	0.001374	1.2388 * (1.2089–1.2695)	0.0157	
	Physical Inactivity	1.1836 * (1.1479–1.2204)	0.0185	0.0003935	1.1840 * (1.1483–1.2209)	0.0188	
	Age (years)	1.1205 * (1.1170–1.1239)	0.0018	0.1101	1.1189 * (1.1155–1.1224)	0.0018	
	Sex (male)	0.9181 * (0.8751–0.9631)	0.0224	0.0001699	1.0570 ^•^ (1.0058–1.1108)	0.0274	
Dia + Hyp	Smoking	1.1600 * (1.0738–1.2532)	0.0458	0.0001883	0.9694 (0.8959–1.0490)	0.0406	0.1130
	Alcoholism	0.6283 * (0.5866–0.6731)	0.0221	0.002696	0.7735 * (0.7200–0.8310)	0.0293	
	Physical Inactivity	1.7353 * (1.6288–1.8488)	0.0561	0.003557	1.5215 * (1.4266–1.6229)	0.0517	
	Age (years)	1.0540 * (1.0500–1.0580)	0.0020	0.02749	1.0539 * (1.0499–1.0579)	0.0020	
	Sex (male)	0.9985 (0.9221–1.0811)	0.0406	0.00000005	1.0733 (0.9890–1.1649)	0.0467	
Dia + Obe	Smoking	0.8691 * (0.7517–1.0049)	0.0646	0.0001209	0.7620 (0.6578–0.8828)	0.0616	0.02958
	Alcoholism	0.8615 (0.7767–0.9555)	0.0456	0.0002661	0.9784 ^#^ (0.8785–1.0896)	0.0567	
	Physical Inactivity	1.5955 * (1.4313–1.7784)	0.0885	0.002072	1.4935 * (1.3393–1.6654)	0.0877	
	Age (years)	1.0576 * (1.0563–1.0589)	0.0007	0.04692	1.0589 * (1.0576–1.0602)	0.0007	
	Sex (male)	1.0960 * (1.0677–1.1250)	0.0146	0.0002526	1.1285 * (1.0981–1.1597)	0.0159	
Hyp + Obe	Smoking	0.9029 * (0.8615–0.9464)	0.0217	0.0000996	0.7309 * (0.6966–0.7669)	0.0184	0.05112
	Alcoholism	1.1615 * (1.1256–1.1986)	0.0186	0.0004555	1.3247 * (1.2813–1.3694)	0.0229	
	Physical Inactivity	1.4697 * (1.4156–1.5259)	0.0281	0.002004	1.3830 * (1.3313–1.4367)	0.0274	
	Age (years)	1.1094 * (1.1058–1.1130)	0.0018	0.09345	1.1081 * (1.1045–1.1117)	0.0018	
	Sex (male)	0.8102 * (0.7674–0.8554)	0.0224	0.0009717	0.9133 * (0.8635–0.9660)	0.0269	
Dia + Hyp + Obe	Smoking	0.8329 * (0.7548–0.9191)	0.0419	0.0002301	0.6940 * (0.6279–0.7669)	0.0372	0.09973
	Alcoholism	0.6518 * (0.6041–0.7032)	0.0253	0.002231	0.8597 * (0.7945–0.9304)	0.0360	
	Physical Inactivity	2.0711 * (1.9371–2.2144)	0.0707	0.006475	1.8685 * (1.7459–1.9996)	0.0669	

^•^ *p* < 0.05, ^#^ *p* < 0.005, * *p* < 0.0005. Abbreviations: Ctl, control; Dia, diabetic; Hth, healthy; Hyp, hypertense; Obe, obese.

## Data Availability

VIGITEL databases is publicly available online at: https://svs.aids.gov.br/daent/cgdnt/vigitel/ (accessed on 1 March 2026).

## Abbreviations

The following abbreviations are used in this manuscript:

BMI	Body Mass Index
Ctl	Control
Dia	Diabetes
Hyp	Hypertension
Obe	Obesity
PA	Physical Activity
PI	Physical Inactivity
VIGITEL	Vigilância de Fatores de Risco e Proteção para Doenças Crônicas por Inquérito Telefônico
